# A case report of atrial septal puncture “mistakenly” penetrated the aorta during left bypass radiofrequency ablation

**DOI:** 10.1097/MD.0000000000037723

**Published:** 2024-04-05

**Authors:** Qian Liu

**Affiliations:** a Department of Cardiology, Shandong Provincial Zibo Central Hospital, Zibo, China.

**Keywords:** accidental puncture into the aorta, radiofrequency ablation, transseptal punctures

## Abstract

**Introduction:**

Transseptal punctures (TSPs) are widely used in left atrium and left ventricle surgery. Accidental puncture of the puncture needle into the aorta is a rare complication that is rarely reported but has serious consequences. The appropriate management of this complication remains unclear.

**Patient concerns:**

This report describes a case of a male with the chief complaint: paroxysmal palpitation for 1 year, aggravated for 1 month.

**Diagnosis:**

The electrophysiological diagnosis was atrioventricular reentrant tachycardia caused by left-side bypass.

**Interventions:**

Radiofrequency ablation of the heart was a necessary treatment and a TSP operation was needed, in which a puncture was mistakenly believed to have entered the aorta, a series of measures were taken urgently. Although the surgical procedure in this case was a false alarm, we still initiated a series of emergency plans. Emergency measures to address the complications were effectively implemented, and the emergency measures were promptly terminated after it was clear that complications had been misjudged.

**Outcomes:**

At last, it was confirmed that the angiogram was a pulmonary artery image, not an aorta image. Then the atrial septal puncture operation was successfully completed, and under the guidance of the Carto system, the ablation was successfully completed. Postoperative fluoroscopy showed no complications, such as pericardial effusion. After 2 years of follow-up, there was no reoccurrence of tachycardia, and there were no complications. It is crucial that emergency procedures are terminated in a timely manner after a clear miscarriage of performance. Although accidental puncture into the aorta is urgent and serious, performing a blockage or even thoracotomy in an emergency if complications are not clearly confirmed can cause further damage to the patient and would be a definitively wrong strategy.

**Conclusion:**

Strict and standardized TSP operations can avoid complications. Correct judgment of the authenticity of complications is crucial, and remedial measures that may cause further damage should not be blindly adopted. The retention of the aortic guide wire can provide convenient access for further differential diagnosis and remedial treatment.

## 1. Introduction

Transseptal punctures (TSPs) are a widely used surgical technique that provide entry into the left atrium (LA) and left ventricle (LV).^[1]^ These include LA and LV catheter radiofrequency ablation, mitral valve interventional surgery, or left atrial appendage closure.^[2]^ The number of surgeries is increasing rapidly.^[3]^ Entering LA through TSP piercing technology requires a certain learning cycle. With strict procedures, this technique is challenging and has inherent risks and safety issues. Its common complications are cardiac perforation and pericardial tamponade and persistent atrial septal defect.^[4–6]^ A rare complication is the accidental insertion of a puncture needle into the aortic root with or without a dilator or sheath.^[1]^ Since the introduction of TSP in the 1950s, this rare but frightening complication has been well known. This is because it can easily lead to sudden intraoperative death in a short period of time, especially in medical centers without cardiac surgical support or experience.^[2]^ At present, due to the rarity of this complication, reports of accidental puncture into the aorta during TSP remain rare, and the appropriate management of this complication remains unclear. This report describes a mistaken assessment of aortic puncture during TSP in a patient with left-side bypass in whom radiofrequency ablation was to be undertaken and the related countermeasures and lessons learned.

## 2. Case description

**Figure 1.**
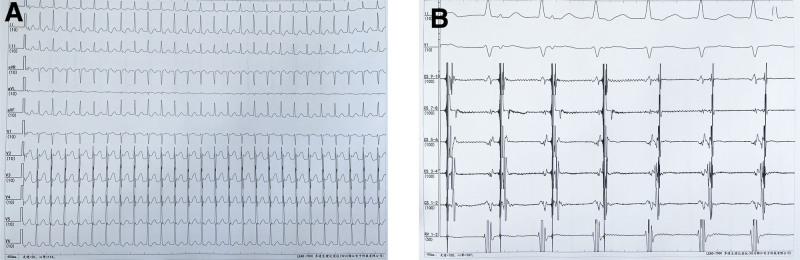
Electrocardiograms. (A) The surface electrocardiogram showed “paroxysmal supraventricular tachycardia.” (B) The electrophysiological electrocardiogram diagnosis was atrioventricular reentrant tachycardia caused by left-side bypass.

Male, 50 years old, chief complaint: paroxysmal palpitation for 1 year, aggravated for 1 month. The patient had palpitations and a sudden stop without obvious cause 1 year ago. It started again a month ago. Electrocardiogram showed “paroxysmal supraventricular tachycardia” (Fig. 1A). He was previously healthy and had no other medical history or surgical history. He had a history of smoking and drinking. Denying a family history of inherited diseases. Physical examination: pulse rate: 65 times/min, blood pressure: 120/80 mm Hg, clear respiratory sound in both lungs, no dry or wet rales, no heart rhythm, and no valve murmurs. The preoperative diagnosis was paroxysmal supraventricular tachycardia.

## 3. Diagnosis and treatment process

**Figure 2.**
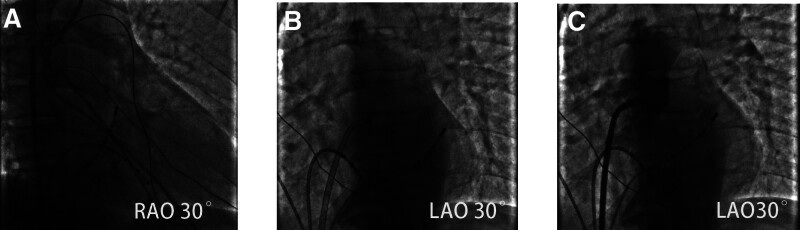
TSP “mistakenly penetrated” into the aorta. (A) Swartz sheath, atrial septal puncture needle reached the height of the superior vena cava. (B) Sent the Swartz sheath along the guide wire. (C) The angiography showed large vessels and sinusoid structures. TSP = transseptal punctures.

The patient gave informed consent. To improve preoperative preparation, the coronary sinus electrode was placed after puncture of the left femoral vein, and the right ventricular quadrupole electrode was placed after puncture of the right femoral vein. The electrophysiological diagnosis was atrioventricular reentrant tachycardia caused by left-side bypass (Fig. 1B). So radiofrequency ablation of the heart was a necessary treatment. The right femoral vein was punctured again, and along the guide wire, the sheath of Swartz (inner diameter 8.5F, S0856332L1, Beijing Heart Nop) and the atrial septal puncture needle (N18710E, Beijing Heart Nop) were sent up to the height of the superior vena cava (Fig. 2A). The failure of the first TSP was considered to be caused by the backward position of the puncture point. The position was adjusted for repuncture. The sheath of Swartz, the dilated sheath, and the guide wire were withdrawn to the inferior vena cava, and the guide wire was sent again; this time, the guide wire went out of the heart shadow. In light of previous structural heart disease, such as atrial septal defect and patent foramen ovale closure, sometimes the guide wire can reach the LA directly from the right atrium through the defect or open septum; thus, the TSP process can be canceled. At this point, the doctor believed that this was possible and sent the dilated sheath and the Swartz sheath along the guide wire (Fig. 2B). Then, angiography was performed using a 20 mL syringe through the Swartz sheath. The angiographic results were not clear, but they still showed that the large blood vessels were out of shape, and the sinusoid structure of the large blood vessels was clearly visible (Fig. 2C).

At this point, the first reaction of the doctor was that the guide wire had been strayed into the aortic root and the Swartz sheath had been pushed into the aortic root. As we all know, this is the most serious complication! In addition, the same or similar events had not occurred in previous surgical cases in our heart center, so we lacked experience in detecting and managing such complications. Thus, in a tense and hurried atmosphere, emergency procedures were immediately initiated. We first left the guide wire in the aorta to keep the sheath in place to prevent forward or backward movement. Heparinization was not administered because the risk of bleeding was not clear. We immediately called the cardiac color ultrasound department, the structural heart surgery team, and the cardiac surgery team and contacted the comprehensive operating room to prepare for possible open-heart surgery. Fortunately, the patient’s vital signs remained stable, giving us plenty of time to prepare.

**Figure 3.**
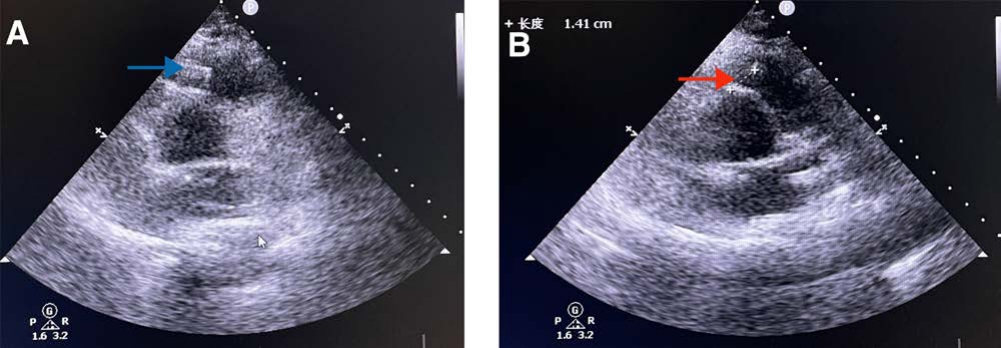
Transthoracic color short axis section of the great artery. (A) Location of the sheath in the right ventricular outflow tract (blue arrow). (B) The nearest distance from the sheath to the right coronary sinus is 1.41 cm (red arrow).

Transthoracic color ultrasonography revealed the following: the position of the sheath in the right ventricular outflow tract was clearly visible from the short axis section of the great artery (Fig. 3A, blue arrow), and the nearest distance from the sheath to the right coronary sinus was measured on this section at 1.41 cm (Fig. 3B, red arrow). After discussion between the structural heart surgery group and the cardiac surgery group, it was concluded that the occlusion operation at this distance might seriously affect the function of the aortic valve; so thoracotomy suture surgery was recommended as a first choice, even though it would involve greater surgical incision trauma and higher surgical risk. However, to avoid more serious complications, thoracotomy suture surgery was still the best option.

**Figure 4.**
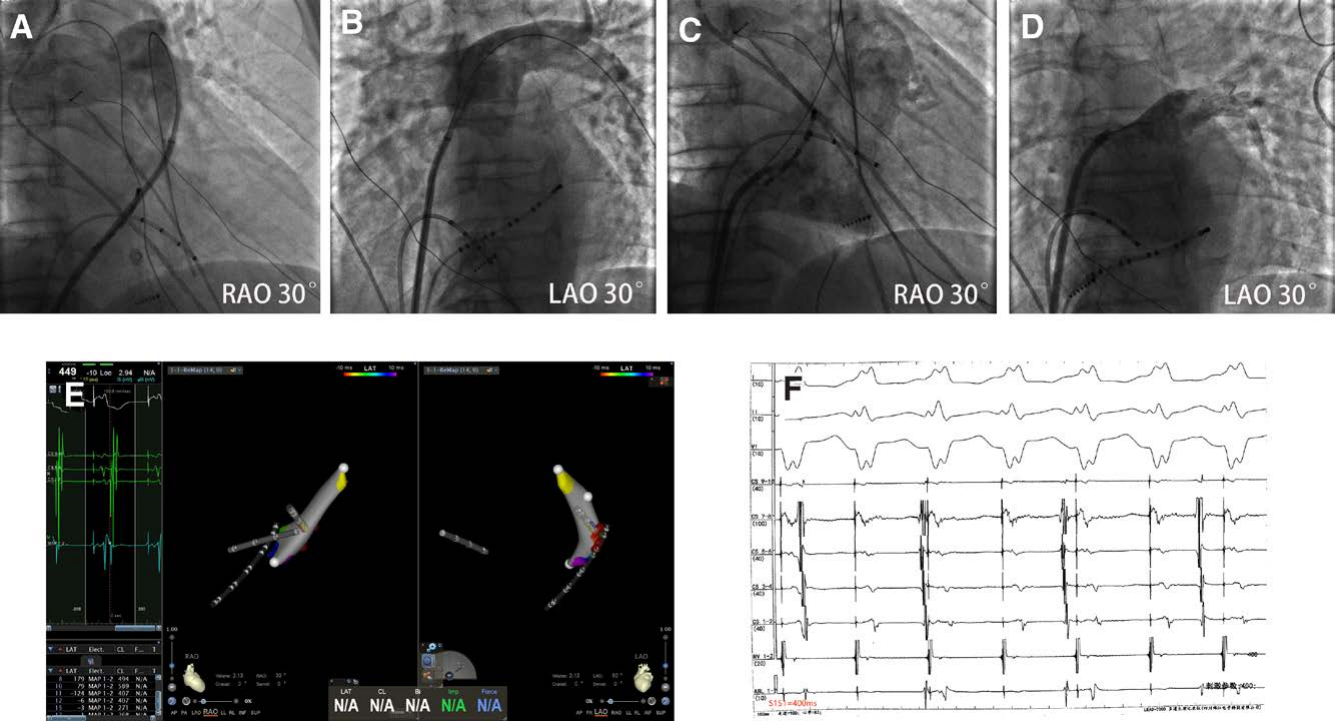
Verification of complications and ablation. (A) The pulmonary artery was shown by angiography (RAO 30°). (B) The pulmonary artery was shown by angiography (LAO 30°). (C) The sheath is located at the lower level of the right atrium, and the angiography shows the right cardiac system. (D) The transseptal puncture operation was completed according to the steps, and angiography showed left atrium and pulmonary veins. (E) The posterior septal bypass was successfully ablated under 3D guidance. (F) Pacing the right ventricle at S1S1 = 400 ms, the electrocardiogram showed the separation of the V and A waves. 3D = 3-dimensional, LAO = left anterior oblique, RAO = right anterior oblique.

No pericardial effusion or pericardial tamponade was found after repeated examination by color ultrasound. Surprisingly, the location of the sheath in the aorta was not clear, and the patient’s vital signs were stable. In view of this, some people in the discussion group suggested that the sheath might not be in the aorta! To ensure safety, we reverified the following: the blood drawn was dark, indicating venous blood; when the hemostatic valve was opened, the blood flow rate showed a low-pressure venous flow rate; connected to the pressure sensor, the pressure waveform was observed, it was from the right heart system; sending the sheath along the guide wire to a higher position for angiography showed that the pulmonary artery was out of shape (Fig. 4A and B). At this point, we determined that the sheath was located in the right cardiac system but not in the left cardiac system. There were still 2 possibilities: the sheath originally located in the aorta was withdrawn into the right cardiac system; the sheath was originally located in the right cardiac system. Combined with the results of cardiac color ultrasound, the discussion group reviewed the angiography results again and again: the large blood vessels shown by the angiography were out of shape and gradually thinning; the images were not images caused by insufficient pressure of contrast agent or dispersion of contrast agent in the aorta, but the blood vessels themselves, that is, the pulmonary sinus and pulmonary artery were out of shape (Fig. 2C). At this time, we had a high probability of determining that the second case applied, that is, the sheath was originally located in the right cardiac system. Under the premise of close monitoring of vital signs, the guide wire was fixed, the sheath was withdrawn, and the vital signs did not change substantially, which further confirmed our judgment that the sheath was originally located in the right cardiac system. The guide wire was retained, the sheath was retracted to the right atrium level, and angiography again showed the right cardiac system and pulmonary artery (Fig. 4C). It was confirmed that the first angiogram was a pulmonary artery image, not an aorta image. At this point, all clear! Then, according to the conventional operation procedures, the long sheath and puncture needle were first sent along the guide wire to the superior vena cava and slowly pulled down, and the puncture point height was determined according to the coronary sinus catheter. At right anterior oblique 30°, the anterior and posterior positions of the puncture points were determined, the atrial septal puncture operation was successfully completed, and the contrast agent at the end of the puncture needle was sprayed into the LA. Left anterior oblique 45° imaging showed clear LA and pulmonary vein contours (Fig. 4D). Under the guidance of the Carto system, the 3-dimensional electrical anatomical structure of the mitral ring was constructed and proved to be the posterior septal bypass, and ablation was successfully completed, then pacing the right ventricle at a frequency of S1S1 = 400 milliseconds, the electrocardiogram showed the separation of the V and A waves (Fig. 4E and F). Postoperative fluoroscopy showed no complications, such as pericardial effusion. After 2 years of follow-up, there was no reoccurrence of tachycardia, and there were no complications.

## 4. Discussion

### 4.1. Further procedure after accidental puncture into the aorta

After TSP puncture, ensure that the puncture needle tip is in the LA before advancing the dilating sheath and long sheath. Angiography was performed at the end of the needle tip, and the guide wire shadow was confirmed to run out of the heart shadow to indicate the puncture into the LA; LA pressure and aortic pressure can be distinguished by measuring needle tip pressure. It has been reported that if only the needle lip enters the aorta, removing the needle often does not cause serious complications; however, if the sheath has entered the aorta, it must not be withdrawn blindly, as catastrophic complications may occur.^[7]^ It has been reported that the dilator sheath and sheath should be pushed following the guide wire immediately by 3 to 4 cm to close the puncture site and prevent massive tamponade,^[2]^ buying time for further remedial measures such as sealing or surgical thoracotomy.

### 4.2. Whether anticoagulation should be performed after accidental puncture of the aorta

In general, heparinized anticoagulation should be administered as soon as possible after a device such as a guide wire or sheath enters the left cardiac system. Some patients may continue to take vitamin K antagonists before surgery or stop anticoagulants only before surgery and bridge with low molecular weight heparin. It has been reported that in the event of an accidental aortic puncture, heparin was not administered during subsequent remedial treatment, but thrombosis was not detected.^[1]^ Since the complications themselves may lead to acute massive pericardial tamponade or sudden intraoperative death,^[2]^ it may not be argued that giving heparin is an effective preventive measure. It was also reported that no pericardial effusion was found on imaging after accidental aortic puncture, and the patient’s vital signs were stable. Given an injection of protamine sulfate, a thrombus was subsequently found in the tip of the guide wire in the aorta; this also induced the formation of anterior descending coronary artery thrombosis and then led to ST-segment elevation myocardial infarction.^[8]^ It is recommended that using protamine sulfate to reverse heparinization should be avoided. However, the removal or advancement of the guide wire, dilator sheath, and sheath in the artery should be carried out slowly to minimize potential thrombosis and shedding. If there is no evidence of active bleeding, continuous heparinization for low activated clotting time levels may be the best strategy to minimize the risk of aortic thromboembolism.^[8]^

### 4.3. Lessons learned from this case

Perforation into the aorta is a rare complication of TSP. Although the surgical procedure in this case was a false alarm, we still initiated a series of emergency plans. Emergency measures to address the complications were effectively implemented, and the emergency measures were promptly terminated after it was clear that complications had been misjudged. The reasons for this are as follows: the patient had a very short isthmus of the tricuspid valve, which allowed the guide wire and sheath to easily ascend to a higher position of the pulmonary artery. The TSP procedure specification was not followed. Radiofrequency ablation in LA or LV often does not involve the congenital defect as the operating channel, even if there is a congenital atrial septal defect or patent foramen ovale and the guide wire can pass through the atrial septum smoothly, because it often does not meet the requirements of radiofrequency ablation. Therefore, the TSP must be carried out in strict accordance with the operating specifications. No angiography or needle tip pressure determination was performed before the forward delivery of the dilated sheath and sheath to evaluate safety. Because the sheath was very high, exceeding the height of the pulmonary valve and similar to the height of the aortic root, and the angiography showed a shape similar to the aortic, we did not correctly identify the pulmonary artery or the aorta from the image. The doctor was extremely nervous and did not further confirm or analyze whether the sheath was in the aorta by blood color, needle pressure, or multiposition angiography. Retaining the guide wire is a very correct operation, providing a convenient way for further differential diagnosis and remedial treatment measures.

The complications of accidental puncture into the aorta during TSP have never occurred in our cardiac center, but we are well aware of its serious consequences although lacking experience, including that of psychological management. In dealing with this critical situation, a doctor’s appropriate mental state is crucial to avoid miscalculation and unnecessary harm. Although we did not actually puncture the aorta, we initiated a series of measures in accordance with emergency procedures. It is also crucial that emergency procedures are terminated in a timely manner after a clear miscarriage of performance. Although accidental puncture into the aorta is urgent and serious, performing a blockage or even thoracotomy in an emergency if complications are not clearly confirmed can cause further damage to the patient and would be a definitively wrong strategy. It is extremely important that we retain the wire throughout the process, regardless of which remedy is used, to provide more favorable protection and easy access.

Traditionally, TSP surgery can be performed only under fluoroscopic guidance. In recent years, transoesophageal ultrasound technology and intracardial ultrasound technology have also been widely used in TSP operations.^[9,10]^ Using these technologies is conducive to reducing the occurrence of complications, but it increases healthcare costs, and we do not make it a requirement. With the development of medical technology, an increasing number of medical centers will use this technology, and the accumulation of necessary experience is conducive to the learning and promotion of TSP technology.

## 5. Conclusion

Strict and standardized TSP operations can avoid complications. When there are abnormalities in the process of puncture, the authenticity of complications should be correctly judged, the specific damage caused by complications should be evaluated in time, and further damage should not be caused by blind remedial measures. The retention of the aortic guide wire can provide convenient access for further differential diagnosis and remedial treatment.

## Author contributions

**Conceptualization:** Qian Liu.
